# Urinary Tract Infections: Prevention, Diagnosis, and Treatment

**DOI:** 10.3390/jcm12155058

**Published:** 2023-08-01

**Authors:** Amelia Pietropaolo

**Affiliations:** 1 Urology Department, University Hospital Southampton NHS Foundation Trust, Southampton SO16 6YD, UK; ameliapietr@gmail.com; 2 European Association of Urology-Young Academic Urologists (EAU-YAU) Urolithiasis and Endourology Working Group, NL-6803 AA Arnhem, The Netherlands

Urinary tract infections (UTIs) are common pathologies that can affect patients of every age and background. The role of the urological community is often to diagnose them and treat them, but also to identify strategies to prevent them. Good collaboration between urologists and microbiologists is the key to finding an effective strategy for UTI treatment. This is even more true in case of more vulnerable individuals, such as pregnant women or children. Several clinical conditions can also increase the risk of UTIs and, in some cases, can cause enhanced morbidities related to them [1]. These are often related to immunosuppression, urinary diversion, diabetes, and neurological pathologies. The prevention of UTI recurrence in these complex cases is difficult, and innovative strategies are needed in order to optimize our treatment options. Multiple previous studies have investigated the etiology of UTIs and described effective tools for their prevention and treatment [2]. The aim of this Special Issue of the *Journal of Clinical Medicine*, with the research topic “Urinary Tract Infection: Prevention, Diagnosis and Treatment”, is to explore different aspects of the prevention and treatment of UTIs, thereby suggesting possible solutions to tackle them in each of their manifestations. With this in mind, an overview of seven original and review articles is included in this Special Issue.

It is known that 50–60% on pregnant women can be affected by UTIs. If not correctly diagnosed and treated, this can lead to clinical issues for the mother or the fetus. Corrales et al. conducted a review of the literature, analyzing all the guidelines currently available for UTI treatment during pregnancy [3]. They also identified families of antibiotics deemed to be safe and ranked them according to their effectiveness. Screening for urinary infections is usually carried out between the 12th and 16th week of gestation in most countries. The first-line antibiotic regime varies among countries, but the most common are Nitrofurantoin, Co-amoxiclav, and first/second- or third-generation Cephalosporin class antibiotics. In cases of acute pyelonephritis, the same antibiotics are used, but with a strong preference for intravenous stewardship.

In later stages of life, pediatric patients are not often affected by urinary tract infections. However, kidney stones disease (KSD) is a known risk factor for recurrent UTIs in this age category. Recent studies have shown that Vitamin D supplementation may be a risk factor for an increased risk of kidney stones in children and, consequently, an increased incidence of UTIs [4]. Some rare clinical conditions and anatomical abnormalities can also increase the risk of infection in this population. Vesicoureteral reflux (VUR) is a rare pathological condition (1–2% of all children) that causes reflux of urine from the urinary bladder to the ureter up to the urinary collecting system. This is often cause of hospital admission in young children due to the high risk of urinary infection, fever, and pyelonephritis, which can lead to renal failure in the long term. An interesting paper by Colceriu et al. [5] describes how the inflammatory process and the immune response in this scenario can sometimes be harmful, leading to increased susceptibility to UTIs and development of tissue damage, scar tissue, and chronic kidney disease. A better understanding of the immunological mechanism of this pathology could help us to explain the cause of reflux nephropathy and to prevent and treat this rare disease.

A recent issue of the modern world related to immune system response and empirical stewardship of antibiotics is also the phenomenon of antibiotic resistance. This leads to a relevant economic and social burden, and some countries are more affected than others. Manolitsis et al. described in their paper that Greece is a country with a high incidence of multidrug-resistant bacteria due to excessive consumption of over-the-counter antibiotics, confirming the country as one of the top four antibiotic prescribers in Europe [6]. Over a time span of 2 years, 12,215 urine and blood samples from patients hospitalized in the urology department were processed by the Microbiology Laboratory. Numerous bacteria were tested, confirming a mean resistance rate of more than 22% to Escherichia Coli and of more than 46% to Klebsiella Pneumoniae. A new computerized model that uses artificial intelligence and machine learning to predict sensitivity to antibiotics will be able to shorten the speed of the diagnostic process, hence allowing for early treatment of UTIs [7]. This study will be useful for organizing stewardship programs that aim to optimize the appropriate use of available antimicrobial agents with better criteria to prevent the development of resistance.

In the urological field, most urinary infections are associated with indwelling devices such as catheters, ureteric stents, and different urinary diversion systems. Catheter-related UTIs are very common, and they are often responsible for high morbidity and costs to the health system worldwide [8]. Jo et al. described that E. Coli was the most common species causing UTIs in patients without indwelling catheters, while in catheterized patients, Enterobacter and Enterococcus species were mostly isolated. However, the study showed no difference in resistance rates between the two groups of patients (catheterized vs. not catheterized). Interestingly, in both groups of patients, Fosfomycin had a success rate higher than 50% for UTIs. According to this study, Fosfomycin should therefore be considered as the primary antibiotic for catheterized patients [9].

Unfortunately, in cases of multidrug-resistant recurrent UTIs, no alternative solutions are available to prevent patient morbidity. Intravesical antibiotics have already been identified as a valid alternative to oral or systemic antibiotics for recurrent infection refractories [10]. A recent systematic review by Ong et al. described the effectiveness of using intravesical Aminoglycoside instillations (IVA) for either treatment or prophylaxis of refractory UTIs, achieving successful outcomes in around 80% of patients [11]. The review takes into account 584 patients who underwent intravesical administration of Gentamicin, Amikacin, Tobramycin, Garamycin, Netilmicin, or Neomycin. Moreover, some studies assessed the use of an intravesical Aminoglycoside in combination with Polymyxin. Most patients were affected by neurogenic bladder, or had urinary diversion or indwelling and intermittent catheters. A success rate of 80% was observed in patients who had IVA alone, and this rate was 79.5% in cases of IVA in combination with Polymixin. The side effects were low and patients’ compliance was excellent, with 6.3% discontinuation reported. The paper described how IVA treatment can effectively solve the issue of multi-resistant organisms in the short term thanks to the avoidance of oral intravenous antibiotics. However, to date, no guidelines are available due to the lack of standardization of doses and administration methods.

As previously mentioned, a strong relationship exists between KSD and UTIs, and indeed, stone formers are at a higher risk of developing UTIs over their lifetimes [12]. For this reason, patients with recurrent UTIs and concomitant KSD may benefit from surgical treatment of the stones [13]. The original paper by Ripa et al. analyzed data of 178 consecutive patients with recurrent UTIs, sepsis, or pyelonephritis who underwent ureteroscopy and laser lithotripsy (FURSL) as a treatment for their urinary stones over a 10-year period. The study showed that 88.8% of the patients were infection-free after the treatment [14]. The overall rate of early post-operative UTI recurrences was 6.2%, with a gradual increase over time, mostly related to stone recurrence. Older or diabetic patients tended to have lower chances of being infection-free after the stone treatment. The outcomes of the paper suggest that stone-free status after FURSL could help to reduce UTI recurrence in the majority of patients.

In rare cases, FURSL itself can be followed by infectious complications. The main reason for these rare, but morbid, events can be related to the intrarenal pressure developed during the procedure, which can be a cause of pyelo-venous backflow and bacteremia leading to sepsis [15], with potentially fatal consequences [16]. Villa et al. carried out an original study to identify possible correlations between the use of a ureteric access sheath (UAS) and post-operative infection during FURSL. The UAS is a useful tool utilized during FURSL to reduce intrarenal pressure and to improve both the removal of fragments and the visibility by promoting a constant outflow from the renal cavities [17]. In their study, Villa et al. analyzed 451 surgical FURSL procedures [18]. In the first 24 hours after surgery, fever, sepsis, and septic shock was described in 52 (11.5%), 10 (2.2%), and 6 (1.3%) cases, respectively. Patients’ comorbidities were positively associated with a 10% higher risk of infectious complications, including sepsis and septic shock (*p* < 0.01). However, according to the study, the use of UAS effectively reduced the risk of septic shock (*p* < 0.04), but not necessarily the risk of fever or sepsis.

As Guest Editor, I would like to thank all the authors for their high-quality contributions, which shine new light on the topic of the prevention and treatment of urinary tract infections. I warmly thank the reviewers for their time in professionally reviewing the papers and raising important comments and queries. Special thanks to the JCM editorial team and Shirley Chen for their collective support, perseverance, and assistance.
